# Completed plastome sequence of *Sophora moorcroftiana*, an endemic shrub to the Qinghai-Tibet Plateau, China

**DOI:** 10.1080/23802359.2019.1692713

**Published:** 2019-11-21

**Authors:** Huie Li, Lan Yang, Qian Li, Jiangrong Li

**Affiliations:** a College of Agriculture, Guizhou University, Guiyang, People’s Republic of China;; b Key laboratory of Forest Ecology in Tibet Plateau of Ministry of Education, Tibet Agriculture & Animal Husbandry University, Nyingchi, China

**Keywords:** Chloroplast genome, leguminosae, leguminous plant, phylogeny

## Abstract

*Sophora moorcroftiana*, an endemic Fabaceae species occurred in the Qinghai-Tibet plateau, China, has important economic value in local. Its completed plastome sequence is 148,930 bp in size, and comprises a pair of inverted repeat (IR) regions of 23,787 bp each, a large single-copy region of 83,342 bp and a small single-copy region of 18,014 bp. The GC content of the plastome was 30.2%. A total of 124 genes were identified, comprising 80 protein-coding genes, 36 tRNA genes and eight rRNA genes. There are 94 unique genes, with 15 genes duplicated in the IR regions. Phylogenetic tree shows that plastome of *S*. *moorcroftiana* is most related to that of *S*. *alopecuroides*. This plastome would be helpful for the study of molecular mechanism of photosynthesis, sustainable conservation, genetic improvement of *S. moorcroftiana*.

*Sophora moorcroftiana*, an endemic Fabaceae species occurred in the Qinghai-Tibet plateau (QTP), China, has remarkable ecological value of its extremely strong drought resistance (Guo et al. 2014; Li et al. 2015; Yao et al. 2016), as well as the ornamental value of its pure blue flowers. Besides, its branches are often for fuel, and the young shoots are often for fodder, and the seeds are usually used to the traditional Tibetan medicine in local (Fu et al. 2016; Li et al. 2017). Therefore, *S. moorcroftiana* has important economic value in the QTP.

Plastids are photosynthetic organelles in plant cells, also being called chloroplasts in higher plants. The completed plastome sequence of higher plant is of great significance to the study of the molecular mechanism of photosynthesis, sustainable conservation, genetic improvement, and so on. Thus, the completed plastome sequence of *S. moorcroftiana* was assembled in this study.

Leaves of *S*. *moorcroftiana* were collected from the QTP at 2931 m high in Nyingchi County, Tibet, China (29°27′4″ N, 94°31′0″ E), and the voucher specimen (NC19185) was deposited in the herbarium of Institute of Plateau Ecology, Tibet Agriculture & Animal Husbandry University, China. The genomic DNA was sequenced using the Illumina Hiseq Platform (Illumina, San Diego, CA). A total of 5.5 G raw reads were quality-trimmed using CLC Genomics Workbench v8 (CLC Bio, Denmark), then used for the plastome sequence assembly process. CLC workbench was also used to de novo assemble. Contigs BLAST results showed that the highest hit was the completed plastome sequence of *S. alopecuroides* (GenBank MF156140) (Duan et al. 2019), so this plastome sequence was used as reference for assembling of the completed plastome sequence of *S*. *moorcroftiana* using software MITObim v1.7 (Hahn et al. 2013). De novo assembly combined with Sanger sequencing of PCR products were used to verify the results. The plastome sequence annotation of *S*. *moorcroftiana* was conducted using the program GENEIOUS R11 (Biomatters Ltd., Auckland, New Zealand) (Kearse et al. 2012) comparing with the *S. alopecuroides* plastome. The coding sequences, tRNAs and rRNAs, were further confirmed and, in some cases, manually adjusted after the BLAST searches. The related plastome sequences were aligned and a NJ tree was conducted using the MEGA 6.0 program (Tamura et al. 2013) with a bootstrap value of 1000.

Finally, a total of 814,419 individual reads generated at average coverage of 702.2. The circular plastome is 148,930 bp in size, and comprises a pair of inverted repeat (IR) regions of 23,787 bp each, a large single-copy (LSC) region of 83,342 bp and a small single-copy (SSC) region of 18,014 bp. The GC content of the completed plastome was 30.2%. A total of 124 genes were identified, comprising 80 protein-coding genes, 36 tRNA genes and eight rRNA genes. There are 94 unique genes, with 15 genes duplicated in the IR regions. This plastome sequence is deposited under accession number MN508806 in Genbank.

Phylogenetic analysis result shows that plastome of *S*. *moorcroftiana* is most related to that of *S*. *alopecuroides*, with a bootstrap support value of 100% (Figure 1). This plastome would be helpful for the further study of sustainable conservation and genetic improvement of *S. moorcroftiana*.

**Figure 1.**
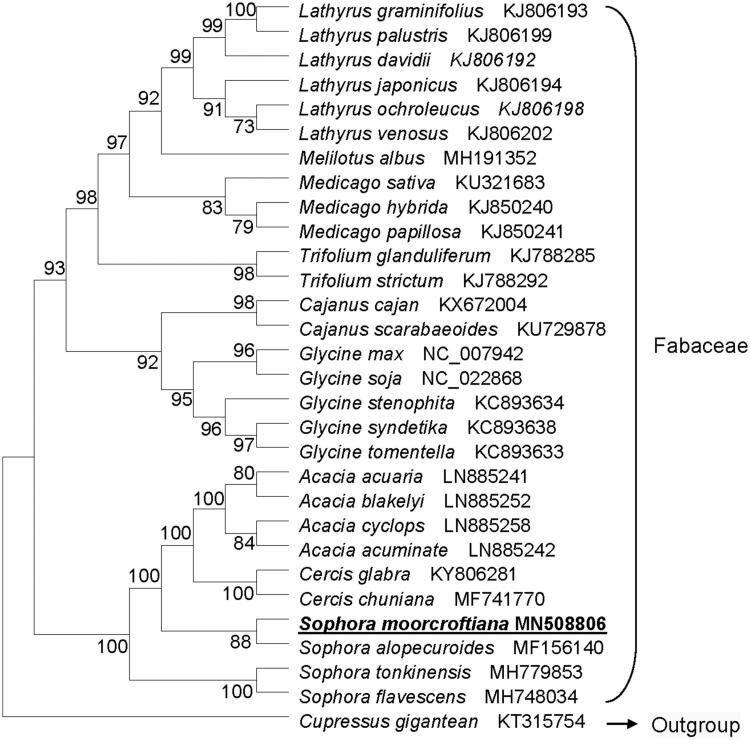
Phylogenetic tree based on the maximum-likelihood (ML) of 30 completed plastome sequences. The bootstrap value based on 1000 replicates is shown on each node. The position of *Sophora moorcroftiana* is shown in bold and underlined.
